# Novel Trispecific Neutralizing Antibodies With Enhanced Potency and Breadth Against Pan‐Sarbecoviruses

**DOI:** 10.1002/mco2.70191

**Published:** 2025-04-21

**Authors:** Rui Qiao, Yuanchen Liu, Qiyu Mao, Jiayan Li, Yinying Lu, Jialu Shi, Chen Li, Jizhen Yu, Jiami Gong, Xun Wang, Yuchen Shao, Lei Sun, Wenhong Zhang, Hongjie Yu, Hin Chu, Pengfei Wang, Xiaoyu Zhao

**Affiliations:** ^1^ Shanghai Sci‐Tech Inno Center for Infection & Immunity National Medical Center for Infectious Diseases Huashan Hospital Institute of Infection and Health Shanghai Key Laboratory of Oncology Target Discovery and Antibody Drug Development Fudan University Shanghai China; ^2^ Shanghai Pudong Hospital State Key Laboratory of Genetic Engineering MOE Engineering Research Center of Gene Technology School of Life Sciences Shanghai Institute of Infectious Disease and Biosecurity Fudan University Pudong Medical Center Fudan University Shanghai China; ^3^ Department of Microbiology School of Clinical Medicine Li Ka Shing Faculty of Medicine The University of Hong Kong Pokfulam Hong Kong Special Administrative Region Hong Kong China; ^4^ Shanghai Fifth People's Hospital Shanghai Institute of Infectious Disease and Biosecurity Institutes of Biomedical Sciences Fudan University Shanghai China; ^5^ Department of Infectious Diseases Shanghai Key Laboratory of Infectious Diseases and Biosafety Emergency Response National Medical Center for Infectious Diseases Huashan Hospital Fudan University Shanghai China; ^6^ School of Public Health Key Laboratory of Public Health Safety Fudan University Ministry of Education Shanghai China

**Keywords:** coronavirus, sarbecovirus, SARS‐CoV‐2, bispecific antibody, trispecific antibody, broadly neutralizing antibody

## Abstract

The ongoing emergence of new variants of the severe acute respiratory syndrome coronavirus 2 (SARS‐CoV‐2) underscores the urgent need for developing antivirals targeting both SARS‐CoV‐2 variants and related sarbecoviruses. To this end, we designed novel trispecific antibodies, Tri‐1 and Tri‐2, engineered by fusing the single‐chain variable fragments (scFvs) of a potent antibody (PW5‐570) to the Fc region of “Knob‐into‐Hole” bispecific antibodies (bsAbs) composed of two distinct broad antibodies (PW5‐5 and PW5‐535). Compared with the parental antibodies, Tri‐1 and Tri‐2 displayed enhanced binding affinities to the receptor‐binding domains of SARS‐CoV, SARS‐CoV‐2 wild type, and Omicron XBB.1.16, with each arm contributed to the overall enhancement. Furthermore, pseudovirus neutralization assays revealed that Tri‐1 and Tri‐2 effectively neutralized all tested SARS‐CoV, SARS‐CoV‐2 variants, and related sarbecoviruses (Pangolin‐GD, RaTG13, WIV1, and SHC014), demonstrating potency and breadth superior to any single parental antibody. Consistently, Tri‐1 and Tri‐2 were found to effectively neutralize authentic SARS‐CoV and SARS‐CoV‐2 variants with IC_50_ values comparable to or better than those of parental antibodies. Taken together, this study highlights the potential effectiveness of Tri‐1 and Tri‐2 as novel formats for harnessing cross‐neutralizing antibodies in the development of multivalent agents to combat both current and future SARS‐like coronaviruses.

## Graphical abstract

Multispecific antibodies harness the advantages of two or more mAbs. In this study, we designed novel trispecific antibodies, Tri‐1 and Tri‐2, derived from our previously identified mAbs, PW5‐5, PW5‐535, and PW5‐570. These trispecific antibodies exhibited enhanced binding affinity and broad neutralization capacity against SARS‐CoV, SARS‐CoV‐2 variants, and related sarbecoviruses, demonstrating potent pan‐sarbecovirus potential with improved efficacy over their parental antibodies.

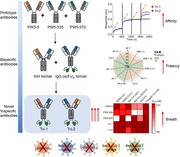

## 1 Introduction

Monoclonal antibodies (mAbs) offer significant promise as therapeutics targeting virus infections, including coronaviruses [1, 2]. Numerous studies have identified and analyzed highly effective neutralizing mAbs against SARS‐CoV‐2, such as LY‐CoV1404 [3], ADG‐2 [4], REGN10933, and REGN10987 [5]. Most of these antibodies specifically target the receptor‐binding domain (RBD) located on the spike (S) glycoprotein of the SARS‐CoV‐2 [6, 7], which facilitates binding to the angiotensin‐converting enzyme 2 (ACE2) receptors on the host cell membranes [8]. The primary mechanism for these mAbs‐mediated neutralization is the blockade of the interaction between the RBD and the ACE2 receptor [9, 10]. However, progressive evolution persists as the virus stabilizes into an endemic epidemiological phase across human communities, leading to the accumulation of numerous mutations in the S protein [11, 12]. The dominantly circulated SARS‐CoV‐2 Omicron variants include descendants of the recombinant XBB lineage, such as XBB.1.16, EG.5.1, and HK.3, as well as descendants of BA.2, including JN.1 and KP.3.1.1 [13, 14]. In particular, JN.1 S contains a concerning accumulation of more than 30 mutations compared to that of BA.2 and has spread rapidly worldwide [13, 15]. More importantly, these circulating SARS‐CoV‐2 variants have shown significant to complete resistance to neutralization by most authorized mAb treatments [13, 16, 17]. Therefore, the potential for antibody resistance resulting from the emergence of virus escape mutants is another concern for the development of mAb‐based treatments, highlighting the importance of identifying broadly neutralizing antibodies that effectively target all the diverse variants of SARS‐CoV‐2.

Bispecific antibodies (bsAbs) or multispecific antibodies integrate the antigen‐binding domains of two or more mAbs into a single framework, offering the advantage of preventing escape mutant emergence without the need for producing multiple mAbs for cocktail therapy [18, 19]. The strategy of utilizing bsAb and multispecific antibodies has demonstrated effectiveness in treating cancer, inflammatory disorders, and viral infections [20, 21]. For instance, a recent study found that escape mutants quickly emerged during the passaging of replicating VSV‐based SARS‐CoV‐2‐S virus in the presence of individual mAbs, whereas treatment of an antibody cocktail with non‐competing effect did not result in the generation of escape mutants [22]. Moreover, bsAbs have attracted a significant amount of interest over the past decade. By the end of 2023, a total of 14 bsAbs have been approved, with 11 indicated for cancer therapy and three for non‐oncology purposes [23]. The application of the bsAbs strategy has been successful in treating cancer [24], autoimmune diseases, inflammatory diseases [25, 26], and viral infectious diseases, including human immunodeficiency virus [18, 27], influenza virus [28, 29], hepatitis B virus [30], dengue virus [31], and Ebola virus [32]. Compared to their individual parental mAbs, the potency of these bsAbs with two distinct viral targets consistently exceeds that of their parental mAbs [33, 34]. This information strongly supports the notion that the rational design of antibodies with non‐overlapping epitopes may offer a powerful approach to minimize mutational escape in SARS‐CoV‐2 variants and other sarbecoviruses.

To date, several cross‐neutralizing antibodies that show efficacy against both SARS‐CoV‐2 and its emerging variants have been identified, including S309 [35] and SA55 [36]. Similarly, we recently successfully isolated high‐potency, broadly neutralizing mAbs from an individual who immunized with an intensive five‐dose COVID‐19 vaccine regimen [37]. One of these mAbs, named PW5‐570, exhibited potent neutralizing activity against all tested SARS‐CoV‐2 variants preceding the BA.5 variant. Additionally, two other mAbs, named PW5‐5 and PW5‐535, were found to possess pan‐sarbecoviruses potential. Importantly, structural analysis revealed that these antibodies exhibited distinct binding profiles, with PW5‐5 and PW5‐535 binding to separate conserved epitopes hidden within the RBD, while the epitope of PW5‐570 partially overlapped with receptor‐binding motif (RBM) to affect ACE2 binding.

In this study, we employed two mAbs, PW5‐5 and PW5‐535, which exhibit broad neutralizing activity against sarbecoviruses, in combination with another potent neutralizing antibody, PW5‐570, to develop novel trispecific antibodies termed Tri‐1 and Tri‐2. Compared with the parental antibodies, we found that both Tri‐1 and Tri‐2 exhibited enhanced binding affinities to the RBDs of SARS‐CoV, SARS‐CoV‐2 wild type (WT), and Omicron XBB.1.16. Furthermore, pseudovirus neutralization assays revealed that Tri‐1 and Tri‐2 effectively neutralized all tested current SARS‐CoV‐2 variants, SARS‐CoV, and their related sarbecoviruses. More importantly, Tri‐1 and Tri‐2 were found to effectively neutralize all the tested authentic SARS‐CoV and SARS‐CoV‐2 variants. This approach of integrating three mAbs targeting distinct S protein epitopes may sufficiently enhance affinity to counteract reductions in binding and neutralization activity. Our study highlights the potential effectiveness of Tri‐1 and Tri‐2 as novel formats for harnessing cross‐neutralizing antibodies in the development of multivalent agents to combat both current SARS‐CoV‐2 variants and other phylogenetically related sarbecoviruses.

## 2 Results

### 2.1 Development and Characterization of Bispecific Antibodies

**FIGURE 1:**
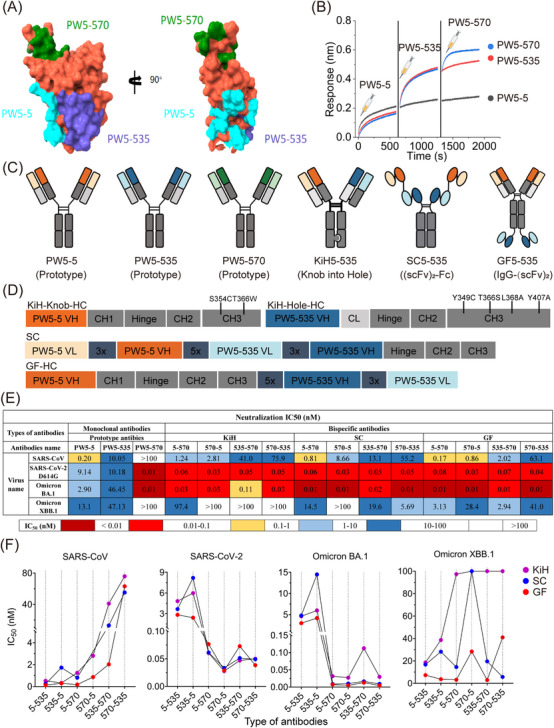
Construction and characterization of different forms of bispecific antibodies. (A) Virtual structure of the prefusion stabilized Omicron XBB S ectodomain trimer in complex with PW5‐5, PW5‐535, and PW5‐570. Surface representation of RBD showing the buried binding site, including PW5‐5 (cyan), PW5‐535 (blue), and PW5‐570 (green). (B) The SARS‐CoV‐2 WT trimer was captured onto the HIS1K biosensors. Then, PW5‐5 was used to saturate the trimer binding sites and the association of PW5‐535 and PW5‐570 was measured sequentially. (C) Schematic diagram of the molecular configurations of prototype antibodies (PW5‐5, PW5‐535, and PW5‐570) and distinct formats of bsAbs. KiH, the Knob‐into‐Hole design with the S354C and T366 W mutations in the heavy chain CH3 region on the Knob arm, and the Y349C, T366S, L368A, and Y407A mutations in the CH3 region on the Hole arm. SC, (scFv)_2_‐Fc format, which is composed of two scFv conjugated to the N‐terminal of the Fc. GF, IgG‐(scFv)_2_, which is composed of one scFv conjugated to the C‐terminal of another antibody IgG1. (D) Schematic presentation of the bsAbs. HC, heavy chain. (E) Heatmap showing the neutralization IC_50_ values of each Ab with indicated pseudotyped viruses. (F) Line chart of bsAbs according to the neutralization IC_50_ values against indicated pseudotyped viruses.

As mentioned above, PW5‐5, PW5‐535, and PW5‐570 exhibited distinct binding profiles. Among them, PW5‐5 and PW5‐535 targeted distinct conserved epitopes within the RBD, whereas PW5‐570 recognized an epitope overlapping the RBM, thereby disrupting ACE2 receptor interactions [37]. To better visualize the binding epitopes of the three antibodies, we simultaneously constructed structures of Omicron XBB RBD in complex with PW5‐5, PW5‐535, and PW5‐570 (Figure 1A). We found that these three antibodies seemed to bind to the Omicron XBB RBD simultaneously, with non‐overlapping epitopes. This finding was further confirmed by in‐tandem BLI binding assays (Figure 1B). Given that these antibodies target distinct epitopes without overlap, it suggests the possibility of combining PW5‐5, PW5‐535, and PW5‐570 into a single molecule to further enhance neutralizing potency and breadth, providing resistance against viral evasion.

As a proof of concept, we constructed various formats of bsAbs to investigate how the choice of format impacts their functionality, including potency and breadth (Figure 1C,D). One format of bsAb is the “Knob‐into‐Hole” format (KiH) with CrossMab design, where the fragment antigen‐binding (Fab) regions of the mAb are swapped. Another format of bsAb involves utilizing a tandem arrangement of scFv domains derived from two mAbs, joined by a (Gly_4_Ser)_5_ peptide linker and subsequently conjugated to the Fc region of human IgG1 comprising hinge‐CH2‐CH3 domains, termed (scFv)_2_‐Fc (SC). In contrast, the other format of bsAb, called IgG‐(scFv)_2_ (GF), uses the heavy chain of IgG1 fused with scFvs with a (Gly_4_Ser)_5_ linker symmetrically. Therefore, a total of six bsAbs of each format were generated based on the random combination of mAbs (PW5‐5, PW5‐535, and PW5‐570) variable regions. All these bsAbs were initially purified using Protein A column affinity chromatography to obtain soluble proteins and further refined by size exclusion chromatography (SEC) to confirm correct folding (Figure S1A–C).

To evaluate the potency and breadth of the bsAbs and their parental mAbs, we next performed neutralization assays using pseudotyped SARS‐CoV, SARS‐CoV‐2 WT, Omicron BA.1, and Omicron XBB.1 viruses (Figure 1E and Figure S2). We found that only bsAbs in the GF format showed neutralization breadth against all tested pseudotyped viruses, whereas corresponding bsAbs in other formats exhibited reduced or complete loss of neutralization activity against Omicron XBB.1 variant in the panel. Furthermore, bsAbs in the GF format exhibited remarkable neutralizing potency against all tested pseudotyped viruses, surpassing that of the corresponding bsAbs in other formats (Figure 1F). Interestingly, the neutralizing activity of the bsAbs GF5‐570 and GF535‐570 against Omicron XBB.1 variant was enhanced approximately 10‐fold compared to the bsAbs GF570‐5 and GF570‐535, indicating that the position of PW5‐570 variable region in the bsAb influences its efficacy.

Overall, these results demonstrate that bsAbs constructed using PW5‐5, PW5‐535, and PW5‐570, with distinct binding epitopes on the SARS‐CoV‐2 S protein, not only maintain the original neutralizing activity but also enhance the breadth of neutralization. Moreover, bsAbs in GF format exhibit superior neutralizing potency and breadth compared to the corresponding bsAbs in other formats.

### 2.2 Novel Trispecific Antibodies Displayed Enhanced Binding Affinity

**FIGURE 2:**
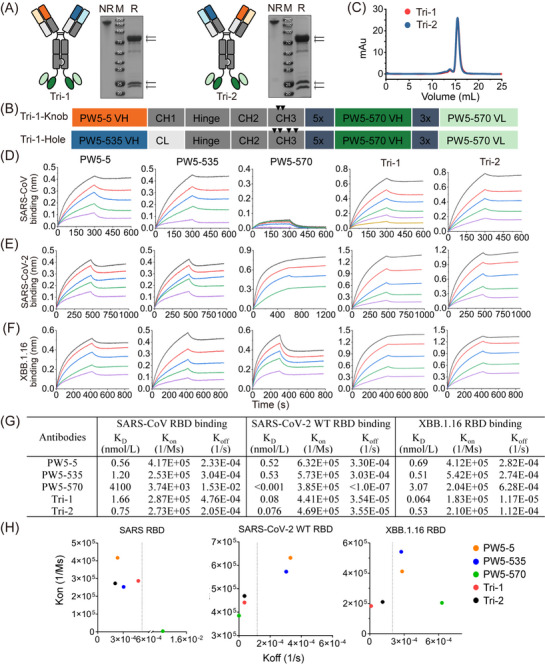
Construction and characterization of novel trispecific antibodies. (A) Schematic diagram of the molecular configurations of trispecific antibodies, Tri‐1 and Tri‐2. Analysis of protein expression by non‐reduced (NR) and reduced (R) SDS‐PAGE. Antibody domains are colored by structural architecture (deep orange and orange, variable heavy and light chain of PW5‐5; deep blue and blue, variable heavy and light chain of PW5‐535; deep green and green, variable heavy and light chain of PW5‐570). (B) Schematic representation of Tri‐1. (C) Purification of indicated antibodies by the size exclusion chromatography (SEC). (D–F) Binding kinetics of trispecific antibodies and prototype antibodies to the RBD of SARS‐CoV (D), SARS‐CoV‐2 WT (E), and XBB.1.16 (F). (G and H) Summary of the data (G) or scatter plot (H) of the affinities (*K*_D_), association (*K*_on_), and dissociation (*K*_off_) of indicated antibodies to the RBDs of SARS‐CoV, SARS‐CoV‐2 WT, and Omicron XBB.1.16, as measured by the bio‐layer interferometry (BLI).

To further improve the neutralizing potency and breadth of the antibodies, we developed a novel trispecific antibody format combining the “Knob‐into‐Hole” CrossMab technology into the GF format (Figure 2A). As shown in Figure 2B, PW5‐570 scFvs were covalently attached to the C‐terminus of the Fc region of KiH5‐535 bsAb and KiH535‐5 bsAb, respectively, yielding two trispecific antibodies, Tri‐1 and Tri‐2. The SDS‐PAGE results showed the correct molecular weight of both trispecific antibodies under both reduced and non‐reduced conditions. Furthermore, the SEC assay confirmed high physical and chemical homogeneity for each trispecific antibody, with a purity exceeding 95% (Figure 2C and Figure S3).

We next evaluated the binding properties of the two trispecific antibodies and their parental antibodies to the RBD proteins of SARS‐CoV, SARS‐CoV‐2 WT, and Omicron XBB.1.16 using BLI‐based kinetic assays (Figure 2D–G). The binding capabilities of Tri‐1 and Tri‐2 to the SARS‐CoV RBD are comparable to those of the two parental antibodies, PW5‐5 and PW5‐535, whereas the parental antibody PW5‐570 has almost completely lost its binding affinity to the SARS‐CoV RBD. In contrast, for the SARS‐CoV‐2 WT RBD, both Tri‐1 and Tri‐2 exhibited over a 10‐fold increase in binding affinity compared to the parental antibodies PW5‐5 and PW5‐535, although PW5‐570 has an excellent binding affinity with *K*_D_ values lower than 0.001 nmol/L. Regarding the Omicron XBB.1.16 RBD, both Tri‐1 and Tri‐2 exhibited high binding affinity with *K*_D_ values in the nanomolar range. Notably, Tri‐1 demonstrated superior affinity compared to the parental antibodies, reflecting a significant decrease of approximately 1–2 log.

For comparison, we also assessed the binding properties of GF5‐570 and GF535‐570, which exhibited the highest neutralizing activity among the bsAbs, to the RBD proteins of SARS‐CoV, SARS‐CoV‐2 WT, and Omicron XBB.1.16 (Figure S4). As expected, GF5‐570 and GF535‐570 showed high binding affinities to all RBDs, but their *K*_D_ values for SARS‐CoV‐2 WT and Omicron XBB.1.16 were increased by about 10 times compared with Tri‐1 and Tri‐2. Meanwhile, we examined the association rate constant (*K*_on_) and dissociation rate constant (*K*_off_) of the trispecific antibodies with the RBD proteins of SARS‐CoV, SARS‐CoV‐2 WT, and Omicron XBB.1.16 (Figure 2H). Compared to the parental antibodies, Tri‐1 and Tri‐2 exhibited lower *K*_off_ for RBDs, particularly for SARS‐CoV‐2 WT and Omicron XBB.1.16. Thus, trispecific antibodies demonstrated higher affinity toward SARS‐CoV‐2 and its variants compared to the parental antibodies, likely due to their multivalent binding to the RBD, resulting in a slower rate of dissociation. Taken together, both trispecific antibodies exhibit binding to the RBD of SARS‐CoV, SARS‐CoV‐2 WT, and Omicron XBB.1.16 with affinity *K*_D_ values in the nanomolar range, which are notably higher or comparable to the binding observed with the precursor mAbs and their GF‐formatted bispecific counterparts.

### 2.3 Tri‐1 Displayed a Significant Synergistic Effect in Binding to the XBB.1.16 RBD

**FIGURE 3:**
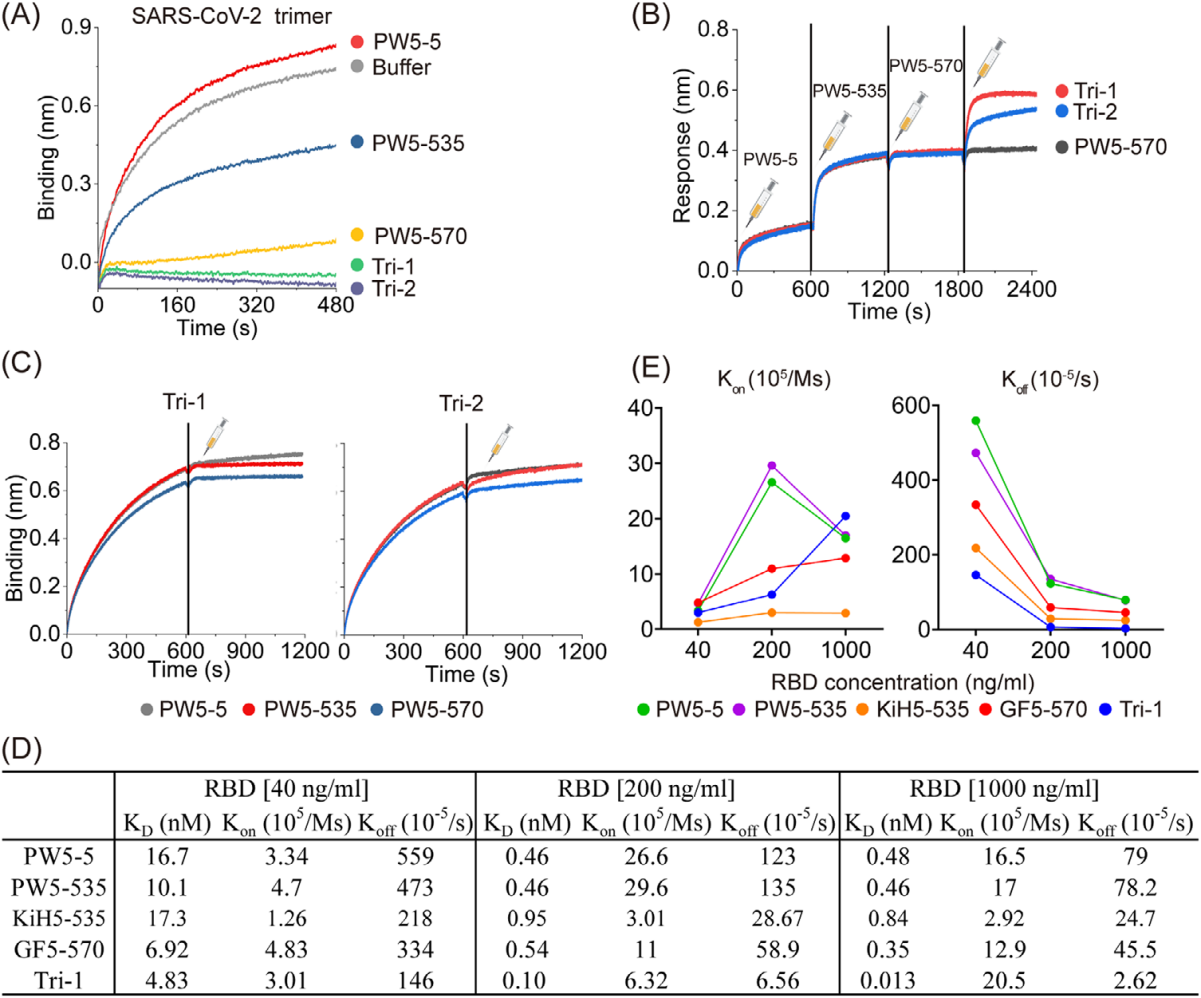
Binding properties of novel trispecific antibodies. (A) Recombinant human ACE2 was immobilized on HIS1K sensors, followed by the addition of a mixture containing the indicated antibodies and SARS‐CoV‐2 WT spike trimer. As a positive control, the antibody was instead by PBST buffer, and then was loaded onto the human ACE2 immobilized biosensor (gray). (B) The XBB trimer was captured onto the HIS1K biosensors. Then, PW5‐5, PW5‐535, and PW5‐570 were sequentially used to saturate the trimer binding sites, and the association of Tri‐1 or Tri‐2 was subsequently measured. (C) The XBB trimer was initially captured onto the HIS1K biosensors, following the indicated prototype antibody (PW5‐5, PW5‐535, or PW5‐570) was injected onto the RBD‐connected biosensors, which have already saturated with Tri‐1 (left) or Tri‐2 (right). (D and E) The XBB.1.16 RBD was captured onto the HIS1K biosensors at 40, 200, and 1000 ng/mL for 300 s, respectively. Summary of the data (D) or scatter plot (E) of the affinities (*K*_D_), association (*K*_on_), and dissociation (*K*_off_) of Tri‐1‐ and structural‐related mAbs and bsAbs to indicated concentrations RBD.

Prior to evaluating the neutralization activity, we first conducted a competition assay using BLI to evaluate the binding of trispecific antibodies to the SARS‐CoV‐2 WT trimer in the presence of ACE2, aiming to elucidate their neutralization mechanism (Figure 3A). Consistent with our previous results, the binding of ACE2 to the SARS‐CoV‐2 WT trimer was indeed suppressed by mixing PW5‐535 and PW5‐570 with the S trimer, while PW5‐5 did not affect this binding. Notably, the mixture of Tri‐1 and Tri‐2 with S trimers further hindered the binding of ACE2 to SARS‐CoV‐2 WT trimers, indicating that a thorough competition between the two trispecific antibodies and ACE2 was more effective than prototype mAbs. We then assessed the binding sites of the trispecific antibodies on the XBB trimer to determine whether their binding sites differed from those of the parental antibodies (Figure 3B). After consecutive saturation with PW5‐5, PW5‐535, and PW5‐570, Tri‐1 and Tri‐2 were still capable of binding to the XBB trimer. This suggests that the two trispecific antibodies may be capable of recognizing additional epitopes or possess a stronger binding effect compared to the parental antibodies. Moreover, both Tri‐1 and Tri‐2 bound to the XBB trimer were able to block the subsequent binding of any parental antibody, suggesting that trispecific antibodies may have much higher affinity than the parental mAbs (Figure 3C).

Next, the binding properties of the Tri‐1 and its corresponding forms to the XBB.1.16 RBD at various concentrations were evaluated in a more detailed manner (Figure 3D). Tri‐1 exhibited the highest binding affinity compared to any other related forms of the antibody, as evidenced by the consistently lower *K*_D_ values among them at different RBD concentrations. When comparing the association rate (*K*_on_) and dissociation rate (*K*_off_) separately, Tri‐1 showed consistently lower *K*_off_ values but not consistently higher *K*_on_ values, indicating that the higher binding affinity of Tri‐1 might be largely attributed to the slower dissociation rate of Tri‐1 compared to other related forms of antibodies (Figure 3D,E). Furthermore, the fold change in *K*_D_ value for Tri‐1 (>300‐fold) between concentrations of 40 ng/mL and 1000 ng/mL is significantly greater than that of any other related antibody form, such as PW5‐5 (35‐fold change), PW5‐535 (22‐fold change), KiH5‐535 (21‐fold change), and GF5‐570 (20‐fold change). Overall, these findings indicate that the significantly enhanced binding effect of Tri‐1 on XBB.1.16 RBD is attributable to the synergistical interaction between PW5‐5 and PW5‐535, as well as the fusion of the PW5‐570 scFv to the C‐terminus of the Fc region.

### 2.4 Novel Trispecific Antibodies Exhibited Enhanced Neutralization Potency and Breadth

**FIGURE 4:**
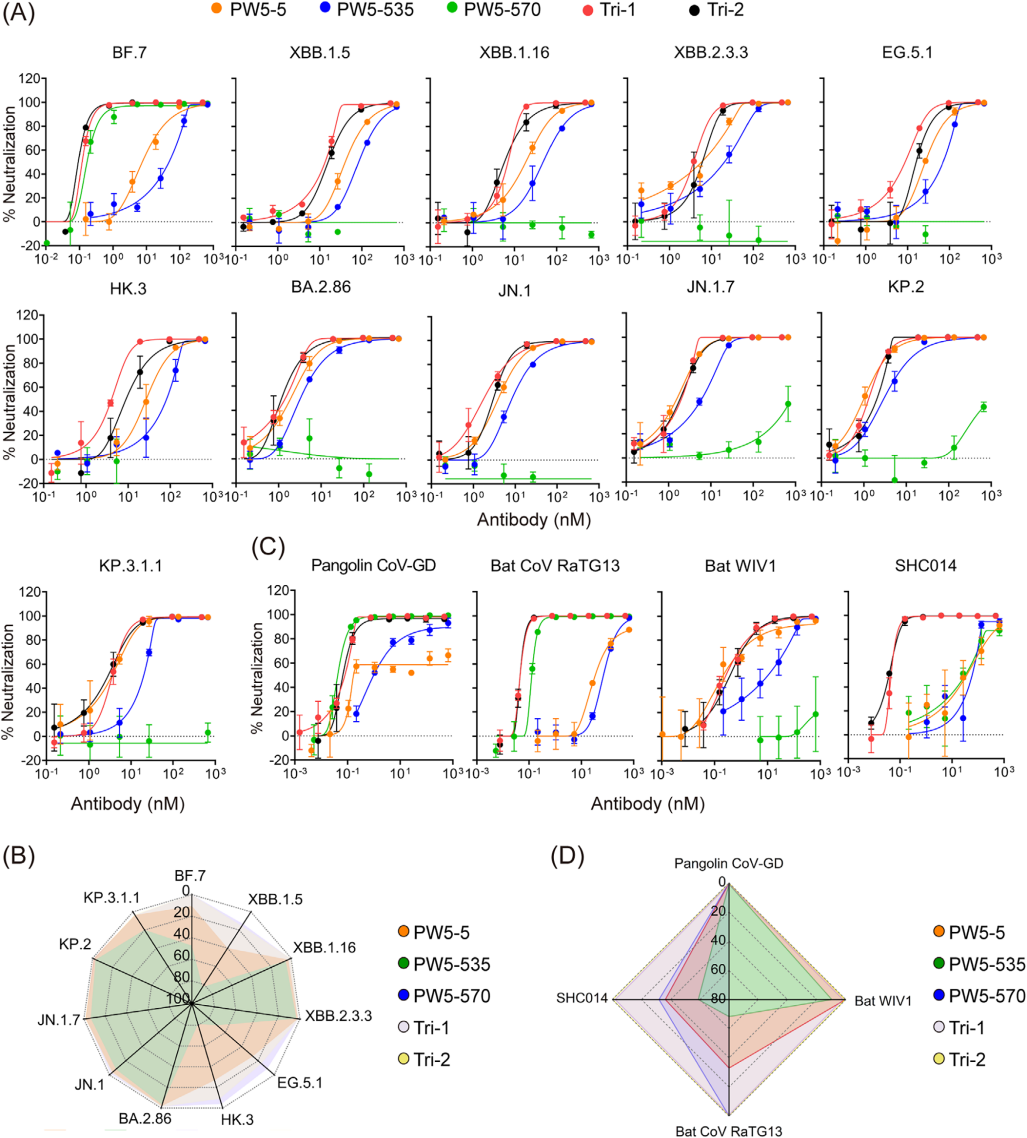
Pseudovirus neutralization of novel trispecific antibodies against the current SARS‐CoV‐2 Omicron variants and other sarbecoviruses. (A) Neutralization by indicated antibodies against the current SARS‐CoV‐2 Omicron variants, including BF.7, XBB.1.5, XBB.1.16, XBB.2.3.3, BA.2.86, EG.5.1, HK.3, JN.1, JN.1.7, KP.2, and KP.3.1.1. (B) Neutralization curves of SARS‐CoV‐2‐ (Pangolin CoV‐GD and Bat CoV RaTG13) and SARS‐CoV‐related sarbecoviruses (Bat WIV1 and SHC014) by indicated antibodies. (C and D) Radar charts were drawn based on the IC_50_ values of indicated antibodies against the current SARS‐CoV‐2 variants (C), and SARS‐CoV‐ or SARS‐CoV‐2‐ related sarbecoviruses (D). All neutralization assays were repeated at least three times and are presented as the mean ± SD.

To assess the potency and breadth of trispecific antibodies and their parental mAbs, we initially performed neutralization assays using various pseudotyped Omicron variants, including BF.7, XBB.1.5, XBB.1.16, XBB.2.3.3, BA.2.86, EG.5.1, HK.3, JN.1, JN.1.7, KP.2, and KP.3.1.1 (Figure 4A). All antibodies exhibited neutralization breadth against all tested SARS‐CoV‐2 Omicron variants, except PW5‐570, which only showed neutralization activity against BF.7 due to the F486 V mutation in the spike protein on other viruses. Moreover, Tri‐1 and Tri‐2 demonstrated enhanced neutralization potency compared to the parental antibodies PW5‐5 and PW5‐535, as evidenced by the 50% inhibitory concentration (IC_50_) values lower than those of PW5‐5 and PW5‐535 (Figure 4B and Figure S5C). We also assessed the neutralization efficacy of GF format bsAbs against these SARS‐CoV‐2 Omicron variants (Figure S5A,C). GF5‐535 and GF535‐5 potently neutralized all Omicron subvariants tested, followed by GF5‐570 and GF535‐570, whereas other bsAbs displayed reduced or complete loss of neutralization activity against certain viruses in the panel. Interestingly, the IC_50_ values of GF5‐570 and GF535‐570 are consistently lower than those of GF570‐5 and GF570‐535, respectively, suggesting that PW5‐570 in the C‐terminus of antibody Fc regions may have a synergistic effect.

The neutralizing capacity of each antibody was further assessed against a panel of four SARS‐CoV‐ and SARS‐CoV‐2‐related sarbecoviruses, all of which possess the ability to utilize human ACE2 as their cellular receptor (Figure 4C and Figure S5C). Consistent with the results for SARS‐CoV‐2 Omicron variants, all antibodies, except PW5‐570, demonstrated neutralization breadth against four SARS‐CoV‐ and SARS‐CoV‐2 related sarbecoviruses (Figure 4D). Among them, Tri‐1 and Tri‐2 showed approximately a 1000‐fold increase in neutralizing potency against SHC014 compared to their parental antibodies (Figure S5C). For pangolin CoV‐GD and bat WIV1 viruses, Tri‐1 and Tri‐2 exhibited comparable or superior neutralizing capabilities compared to their parental antibodies, with IC_50_ values ranging from 0.07 to 0.37 nM.

**FIGURE 5:**
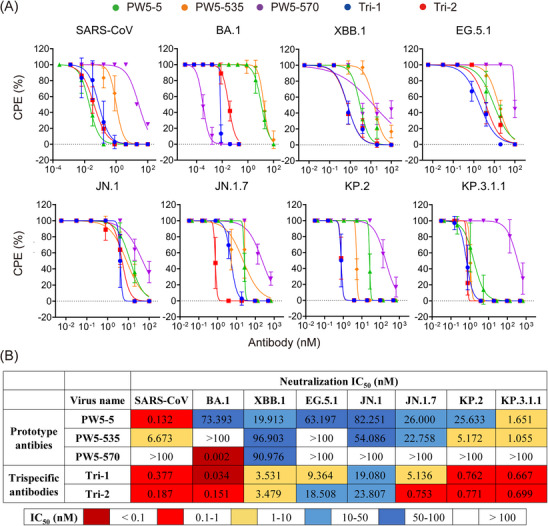
Neutralization of novel trispecific antibodies against authentic SARS‐CoV and SARS‐CoV‐2 variants. (A and B) Neutralization curves (A) and IC_50_ values (B) of trispecific antibodies and their prototype antibodies against authentic SARS‐CoV and major SARS‐CoV‐2 Omicron variants, including BA.1, XBB.1, EG.5.1, JN.1, JN.1.7, KP.2, and KP.3.1.1. The data represent one of at least three independent experiments and are presented as the mean ± SD.

Next, we conducted authentic virus neutralization assays in VeroE6‐TMPRSS2 cells for Tri‐1 and Tri‐2, along with their parental antibodies (Figure 5A). Similar to the pseudovirus results, Tri‐1 and Tri‐2 consistently demonstrated broad neutralization against all tested SARS‐CoV‐2 Omicron variants (BA.1, XBB.1, EG.5.1, JN.1, JN.1.7, KP.2, and KP.3.1.1) and SARS‐CoV with IC_50_ values ranging from 0.034 to 23.807 nM (Figure 5B). In contrast, the neutralization activity of their parental antibodies was reduced or completely lost for certain viruses in this panel. Besides, the neutralizing potency of Tri‐1 and Tri‐2 against XBB.1, EG.5.1, and JN.1 increased approximately 5–20 times, while their potency against SARS‐CoV and BA.1 remained relatively unchanged compared to the parental antibodies. Taken together, these results demonstrated that Tri‐1 and Tri‐2 substantially increased the neutralization potency and extended their neutralizing breadth by recovering the neutralization against SARS‐CoV, SARS‐CoV‐2 variants, and their related sarbecovirus in comparison with the parental mAbs.

## 3 Discussion

Although the COVID‐19 global pandemic caused by SARS‐CoV‐2 has been declared ended by the World Health Organization, the virus continues to evolve through genomic changes induced by genetic mutations or viral recombination, leading to the emergence of variants that differ from the original SARS‐CoV‐2 virus [38, 39]. As a consequence, the efficacy of previously licensed or approved mAbs, such as LY‐CoV016, LY‐CoV555, AZD1061, REGN‐10933, and REGN‐10987, is significantly diminished due to epitopes mutations, particularly those located in the RBD region [40]. This highlights the urgent need to identify broadly neutralizing antibodies capable of effectively targeting a wide range of SARS‐CoV‐2 variants [41].

According to phylogenetic analysis of coronavirus genomes, SARS‐CoV‐2 belongs to the *Betacoronavirus* genus, which also includes SARS‐CoV, bat SARS‐related coronaviruses (SARSr‐CoV), and other coronaviruses found in humans and other animal species. While SARS‐CoV‐2 exhibits a 96.2% genomic sequence identity with its closest relatives, SARS‐CoV and other SARSr‐CoVs demonstrate significantly lower sequence homology, sharing less than 80% identity with SARS‐CoV‐2 [42]. Recent advances have identified several broadly neutralizing antibodies that exhibit cross‐reactivity against SARS‐CoV‐2 and its variant sublineages, including S309 [35] and SA55 [36]. Our team has also recently isolated a panel of broad‐spectrum neutralizing mAbs from an individual who underwent an intensified five‐dose heterologous COVID‐19 vaccination regimen [37]. The lead antibody, PW5‐570, demonstrated ultrapotent neutralization across all SARS‐CoV‐2 VOCs, although with reduced efficacy against BA.5. Notably, two distinct mAbs, PW5‐5 and PW5‐535, exhibited cross‐sarbecovirus activity. Importantly, these antibodies exhibited distinct binding profiles, with PW5‐5 and PW5‐535 binding to separate conserved epitopes hidden within the RBD, while PW5‐570's epitope partially overlapped with RBM to affect ACE2 binding.

The bsAbs represent a novel class of therapeutic agents designed to combine the binding properties of two antibodies within a single molecule, providing the advantage of preventing escape mutant emergence without the need to produce multiple mAbs for cocktail therapy. Moreover, the strategy of utilizing bsAbs has been effectively employed in the management of viral infections by targeting different viral epitopes or both viral and host epitopes, or by recruiting host cellular immune system functions [43]. Currently, various formats of bsAbs have been developed, including “Knob‐into‐Hole” format, (scFv)_2_‐Fc format, IgG‐(scFv)_2_ format, crossover dual variable (CODV) format, dual variable domain (DVD) format, and others [44]. Asokan et al. [27] demonstrated that bsAbs in the CrossMAb format, combining the binding characteristics of two parental antibodies, can efficiently neutralize 97% of viral strains with high overall potency for the prevention and treatment of HIV‐1 infection. Another format of bsAbs for HIV‐1 prophylaxis and therapy, (scFv)_2_‐Fc format, produced by Wu et al. [45], constructed by engineering a bispecific IgG‐scFv through genetic fusion of a gp120‐targeting scFv (PGT128) and a CD4‐directed scFv (Hu5A8) to the Fc domain of human IgG1 using (Gly_4_Ser)_3_ linkers. In addition, the IgG‐(scFv)_2_ format is also a promising approach for developing bsAb against SARS‐CoV‐2 infection [34, 46–48]. To date, a numerous repertoire of bsAbs format is available. Hence, engineering a bsAb that merges the high neutralization potency of PW5‐570 with the broad‐spectrum neutralization capability of PW5‐5 or PW5‐535 into a single molecule may offer further insights into the development of effective bsAb‐based COVID‐19 therapeutics.

In this study, we first constructed various formats of bsAbs using PW5‐5, PW5‐535, and PW5‐570 to investigate how the choice of format impacts their functionality, including potency and breadth (Figure 1). We found that only bsAbs in the GF format showed neutralization breadth against all tested pseudotyped viruses, whereas corresponding bsAbs in other formats showed reduced or abolished neutralization activity for Omicron XBB.1 variant. Furthermore, bsAbs in the GF format exhibited remarkable neutralizing potency against all tested pseudotyped viruses, surpassing that of the corresponding bsAbs in other formats. Interestingly, the neutralizing activity of the bsAbs GF5‐570 and GF535‐570 against Omicron XBB.1 variant was enhanced approximately 10‐fold compared to the bsAbs GF570‐5 and GF570‐535, indicating that the location of PW5‐570 variable region in the bsAb influences its efficacy.

Next, we developed two novel trispecific antibodies, Tri‐1 and Tri‐2, by combining mAbs of PW5‐5, PW5‐535, and PW5‐570, based on the GF format design (Figure 2). We found that both trispecific antibodies exhibit binding to the RBD of SARS‐CoV, SARS‐CoV‐2 WT, and its Omicron variant XBB.1.16 with affinity *K*_D_ values in the nanomolar range, which are notably higher or comparable to the binding observed with the parental antibodies as well as bsAbs in GF format. Moreover, we determined the binding properties of the two trispecific antibodies and related forms of the antibody to the XBB.1.16 RBD at different concentrations (Figure 3). Notably, we found that all the PW5‐5, PW5‐535, and PW5‐570 arms contribute to the significantly enhanced synergistic binding effect of Tri‐1 on XBB.1.16 RBD.

Apart from the binding affinity, we performed the neutralization assays to evaluate the potency and breadth of the trispecific antibodies and their parental mAbs (Figure 4). These assays were performed using pseudotyped SARS‐CoV‐2 Omicron variants, including BA.1, BF.7, XBB.1, XBB.1.5, XBB.1.16, XBB.2.3.3, BA.2.86, EG.5.1, HK.3, JN.1, JN.1.7, KP.2, and KP.3.1.1, as well as four SARS‐CoV‐ and SARS‐CoV‐2‐related sarbecoviruses with confirmed human ACE2 receptor tropism, namely, WIV1, SHC014, Pangolin‐GD, and RaTG13. We found that Tri‐1 and Tri‐2 substantially increased the neutralization activity and extended their neutralizing breadth by recovering the neutralization against SARS‐CoV, SARS‐CoV‐2 variants, and their related sarbecovirus in comparison with the parental mAbs. To a large extent, these SARS‐CoV‐ and SARS‐CoV‐2‐related sarbecoviruses that use human ACE2 as a receptor are neutralized by blocking the RBD‐ACE2 interaction [49, 50], as described that the trispecific antibodies are highly capable of competing against ACE2 binding (Figure 3A). Interestingly, the IC_50_ values of GF5‐570 and GF535‐570 are consistently lower than those of GF570‐5 and GF570‐535, respectively, suggesting that PW5‐570 in the C‐terminus of antibody Fc regions have a better activity. Similar as the pseudovirus results, Tri‐1 and Tri‐2 consistently demonstrated broad neutralization against all tested authentic SARS‐CoV and SARS‐CoV‐2 Omicron variants, including SARS‐CoV, BA.1, XBB.1, EG.5.1, JN.1, JN.1.7, KP.2, and KP.3.1.1, with IC_50_ values ranging from 0.034 to 23.807 nM (Figure 5). In contrast, the neutralization activity of their parental antibodies was reduced or completely lost for certain viruses in the panel. For example, PW5‐5 and PW5‐535 exhibited neutralization activity against the BA.1 pseudotyped virus, but not the live virus. The underlying cause of this discrepancy is likely due to the differences between pseudoviruses and live viruses. Pseudoviruses, unlike live viruses, contain only structural components of the viral spike protein and lack the complete physiological characteristics of live viruses. This fundamental difference may influence the sensitivity of neutralizing antibodies to viral attachment and entry into cells. On the other hand, while PW5‐5 and PW5‐535 demonstrated broad neutralizing capabilities against sarbecoviruses, their effectiveness was lower than that of PW5‐570. As a result, they may be more susceptible to discrepancies between the two virus types, leading to significant differences in neutralization results when comparing live viruses with pseudoviruses.

There are some limitations in our research. First, although Tri‐1 and Tri‐2 demonstrated enhanced neutralization potency compared to the parental antibodies PW5‐5 and PW5‐535 (Figure 4B and Figure S5C), they still showed no activity against pseudotyped MERS‐CoV (data not shown), indicating that their neutralizing effect is limited to pan‐sarbecoviruses. Second, although no evidence showed that Y349C/T366S/L368A/Y407A mutations affect the binding capacity of Fc to associated receptors or alter the Fc effector functions of antibodies, we did not investigate the changes in Fc effector functions of bsAb in our study. Third, compared to conventional IgG format, Tri‐1 and Tri‐2 have a higher molecular weight, which may affect their absorption, distribution, and metabolism in vivo. However, these factors should be considered in future animal protection experiments.

In conclusion, we successfully developed two novel trispecific neutralizing antibodies in this study, providing insights into their design principles, antigen‐binding and neutralizing properties, and the structural basis underlying their enhanced efficacy compared to parental antibodies. Our findings suggest that Tri‐1 and Tri‐2 may serve as candidates for preventing infections caused by sarbecoviruses, potentially playing a pivotal role in combating novel and recurrent coronavirus outbreaks.

## 4 Materials and Methods

### 4.1 Design of Bispecific and Trispecific Antibodies

For the “Knob‐into‐Hole” format, the bsAbs arm was paired with a heterodimeric antibody arm, crossed between the constant light (CL) domain and the constant heavy 1 (CH1) domain of the light chain. In addition, S354C/ T366 W mutations in the “Knob” heavy chain and Y349C/ T366S/ L368A/ Y407V mutations in the “Hole” heavy chain were engineered into the CH3 region. For the “(scFv)_2_‐Fc” format, the antibody variable heavy chain (VH) and variable light chain (VL) are connected with a (Gly_4_Ser)_3_ linker to form the scFv. This scFv is then linked to a heterodimeric antibody scFv using a (Gly_4_Ser)_5_ linker, followed by fusion to the N‐terminus of the human IgG1 Fc regions to generate a bispecific heavy chain. For the “IgG‐(scFv)_2_” format, the antibody VH and VL regions are connected with a (Gly_4_Ser)_3_ linker to form the scFv. This scFv is then fused to the C‐terminus of antibody Fc regions to generate a bispecific heavy chain that pairs with the light chain of another antibody.

For the “novel trispecific antibody” format, KiH and CrossMab technologies were employed to minimize the risk of molecular mismatch in experimental design. In detail, the VH and VL regions of PW5‐570 are connected with a (Gly_4_Ser)_3_ linker to form the scFv. This scFv is then fused to the C‐terminus of the Fc regions of KiH5‐535 bsAb and KiH535‐5 bsAb separately, generating the trispecific antibodies Tri‐1 and Tri‐2. All antibody variable domain genes in this study were codon‐optimized for enhanced expression in human cell systems and cloned into the gWiz vector (Genlantis) containing either human IgG1 heavy chain constant (CH1‐CH3) regions or light chain constant region (CL).

### 4.2 Antibody Expression and Purification

For antibody production, Expi293F cells (ThermoFisher, A14527) were co‐transfected with heavy and light chain plasmids using polyethyleneimine (PEI, Polysciences) and cultured in SMM 293T‐II medium (Sino Biological) under standard conditions (37°C, 125 rpm, 8% CO₂). After 6 days, the supernatants were filtered and subjected to MabSelect PrismA (Cytiva, 17549801) affinity purification. The antibodies were then concentrated and purified through size‐exclusion chromatography on a Superose 6 Increase 10/300 GL column (Cytiva, formerly GE Healthcare Life Sciences) at 0.75 mL/min, with peak fractions collected at 280 nm. Final characterization included Nanodrop concentration measurement (Thermo Fisher) and SDS‐PAGE analysis under reduced and non‐reduced conditions.

### 4.3 Protein Expression and Purification

Plasmids encoding stabilized soluble spike trimers of SARS‐CoV‐2 (S‐2P) and XBB (S‐6P) were constructed and purified as described previously [37]. Briefly, SARS‐CoV‐2 or XBB spike trimer with two or six proline mutations were constructed into expression vector pcDNA3.1 with 8 × His tags or 2 × Strep‐tag II tags at the C‐terminus. The RBDs of SARS‐CoV, SARS‐CoV‐2 WT, and Omicron XBB.1.16 were individually cloned into the pCMV3 mammalian expression vector, incorporating C‐terminal 8× His tags and 2× Strep‐tag II tags for subsequent protein purification and detection. The mammalian expression plasmid encoding recombinant soluble dimeric ACE2 was obtained from Addgene. Expi293 cells (Thermo Fisher Scientific) were transiently transfected with the corresponding expression plasmids using PEI. The supernatants were harvested on Day 6, and then purified using Ni‐NTA resin or Streptactin resin from Smart Lifesciences (Changzhou, China, Ni Smart Beads 6FF: SA036100 and Streptactin Beads 4FF: SA053500) following the manufacturer's protocol.

### 4.4 Production of Pseudoviruses

After synthesizing plasmids encoding the spike protein from SARS‐CoV, SARS‐CoV‐2, its variants, and related sarbecoviruses, pseudoviruses were generated by co‐transfecting HEK‐293T cells with PEI. Following overnight incubation at 37°C with 5% CO₂, the cells were infected with VSV‐G pseudotyped ΔG‐luciferase (G*ΔG‐luciferase, Kerafast, MOI = 5) in DMEM containing 10% fetal bovine serum (FBS). Post‐infection, the cells were washed three times with PBS/2% FBS, and the medium was then replaced with DMEM/2% FBS. After 24 h, supernatants were collected, clarified by centrifugation at 4000 rpm for 10 min, and further incubated with 20% I1 hybridoma supernatant (anti‐VSV‐G; ATCC CRL‐2700) at 37°C for 1 h to neutralize residual VSV‐G pseudotyped virus. The virus stocks were then titrated and stored at −80°C.

### 4.5 Pseudovirus Neutralization Assay

Neutralization activity was conducted using a pseudovirus‐based assay, as previously described [51]. Briefly, Vero‐E6 cells were plated in 96‐well plates at 2 × 10^4^ cells/well. After 24 h, pseudoviruses were pre‐incubated with serially diluted mAbs (triplicate wells) for 30 min at 37°C before application to the cell monolayer. Following a 24‐h incubation, luciferase activity was quantified using the Luciferase Assay System (RG062 M, Beyotime). The IC_50_ value was determined as the antibody dilution yielding 50% reduction in relative luminescence units compared to virus‐infected controls, after subtracting background signal from cell‐only wells. Data were analyzed using nonlinear regression in GraphPad Prism 8.0

### 4.6 Bio‐Layer Interferometry Assay

The binding affinities of the antibodies were determined through BLI experiments conducted on an Octet R8 instrument (Sartorius), following the manufacturer's guidelines. In brief, RBDs of SARS‐CoV, SARS‐CoV‐2 WT, and Omicron XBB.1.16 at a concentration of 10 µg/mL were loaded onto HIS‐1K biosensors, respectively. After a baseline step in PBST for 120 s, the mAbs, bsAbs, and trispecific antibodies serially diluted into six distinct concentrations were exposed to the sensors for 300–420 s and then the biosensor surface was subjected to dissociation in PBST for 300–600 s to monitor antibody dissociation kinetics. For sensors requiring multiple analyte analyses, surface regeneration was performed between experiments using 0.1 M glycine buffer (pH 1.7), followed by neutralization with PBST in three cycles at the end of the binding assay. For curve fitting, at least four consecutive twofold dilutions were used, with *R* ^2^ values measured using the Octet R8 instrument (Sartorius) as a reference standard.

For in‐tandem BLI binding assays, 10 µg/mL SARS‐CoV‐2 WT or Omicron XBB spike trimer was captured on the HIS1K biosensors. Following a 120 s baseline step in PBST buffer, the biosensors were exposed to the first antibody (100 nM) in PBST buffer for 600 s. Subsequently, the second antibodies (100 nM) were introduced for 600 s and so forth. Additional baseline steps between two antibodies were conducted for 15 s in PBST buffer.

For ACE2 competition assay, the HIS1K biosensor was loaded with recombinant ACE2. After a baseline step of 120 s in PBST buffer, it was then exposed to a pre‐incubated mixture of antibody (400 nM) and SARS‐CoV‐2 WT spike trimer (50 nM) for 600 s, followed by blocking in PBST buffer. A SARS‐CoV‐2 WT spike trimer without antibody served as a positive control.

For antibody binding properties assays, the Omicron XBB.1.16 RBD at the concentration of 40, 200, and 1000 ng/mL were loaded onto HIS‐1K biosensors for 300 s, respectively. After a baseline step in PBST buffer for 120 s, the antibodies at various dilutions were exposed to the sensors for 300 s and then dissociated for 420 s into PBST buffer to evaluate interaction kinetics. The *K*_on_, *K*_off_, and *K*_D_ of antibodies with RBDs were calculated by aligning and fitting to a 1:1 binding model and a global fitting model using Octet Analysis Studio 13.0.

### 4.7 Viruses and Biosafety

SARS‐CoV GZ50 (GenBank accession number AY304495) was a previously archived clinical isolate at the University of Hong Kong [52]. Omicron BA.1 (GISAID: EPI_ISL_6841980), XBB.1 (GISAID: EPI_ISL_15602393), EG.5.1 (GISAID: EPI_ISL_18461518), JN.1 (GISAID: EPL_ISL_18841631), JN.1.7 (GISAID: EPI_ISL_19351034), KP.2 (GISAID: EPI_ISL_19351035), and KP.3.1.1 (GISAID: EPI_ISL_19534723) strains were isolated from COVID‐19 patients in Hong Kong. All SARS‐CoV and SARS‐CoV‐2 variants were cultured and titrated using a VeroE6‐TMPRSS2 cell line by plaque assays [53]. Experiments involving live SARS‐CoV and SARS‐CoV‐2 were carried out in compliance with the approved standard operating procedures of the Biosafety Level 3 facility at the University of Hong Kong.

### 4.8 Authentic SARS‐CoV and SARS‐CoV‐2 Variants Neutralization

Antibody neutralization potency was evaluated using an endpoint titration assay [54]. Briefly, VeroE6‐TMPRSS2 cells (2 × 10⁴ cells/well) were seeded in 96‐well plates and incubated overnight. Serially diluted antibodies (*n* = 9 replicates) were pre‐incubated at 37°C for 60 min with SARS‐CoV and SARS‐CoV‐2 variants (BA.1, XBB.1, EG.5.1, JN.1, JN.1.7, KP.2, and KP.3.1.1) before being added to the cell monolayers (100 PFU/well). After 96 h, cytopathic effects were assessed by two blinded observers. Neutralization percentages, relative to virus‐only controls, were plotted against antibody concentrations, and IC_50_ values (mean ± SD) were determined using a seven‐parameter nonlinear regression model in GraphPad Prism 8.0.

### 4.9 Statistical Analysis

IC_50_ and EC_50_ values were determined using nonlinear regression analysis in GraphPad Prism 8.0. Data are presented as means ± SD. The *K*_on_, *K*_off_, and *K*_D_ of antibodies were calculated by fitting the binding data to a 1:1 binding model and applying a global fitting model using Octet Analysis Studio 13.0.

## Author Contributions

X.Z., P.W., and H.C. conceived and supervised the project. R.Q., J.L., Y. Lu, C.L., J.Y., J.G., X.W., and Y.S. conducted the biological experiments. Y. Liu and J.S. performed authentic neutralization assays. Q.M. performed the structure determination based on Cryo‐EM data. R.Q., Y. Liu., L.S., W.Z., H.Y., H.C., P.W., and X.Z. analyzed the results and wrote the manuscript. All the authors reviewed, commented, and approved the manuscript.

## Conflicts of Interest

X.Z., R.Q., J.L., and P.W. are listed as inventors on two patent applications related to the mAbs (PW5‐5, PW5‐535, and PW5‐570) discussed in this article. However, they have no relevant financial or non‐financial interests to disclose. The authors declare no conflicts of interest.

## Ethics Statement

The authors have nothing to report.

## Supporting information



Supporting Information

## Data Availability

The data that support the findings of this study are available from the corresponding author upon reasonable request.
