# Supplementary material for: Novel Trispecific Neutralizing Antibodies With Enhanced Potency and Breadth Against Pan‐Sarbecoviruses
Source: MedComm (2020). 2025 Apr 21;6(5):e70191. doi: 10.1002/mco2.70191 (PMC12010136; doi:10.1002/mco2.70191)
Supplement: Supplementary file 1 — Supporting Information [file MCO2-6-e70191-s001.docx]

**Novel trispecific neutralizing antibodies with enhanced potency and breadth against pan-sarbecoviruses**

Rui Qiao^1,2,#^, Yuanchen Liu^3,#^, Qiyu Mao^4^, Jiayan Li^2^, Yinying Lu^1^, Jialu Shi^3^, Chen Li^2^, Jizhen Yu^2^, Jiami Gong^2^, Xun Wang^2^, Yuchen Shao^2^, Lei Sun^4^, Wenhong Zhang^5^, Hongjie Yu^6^, Hin Chu^3,*^, Pengfei Wang^1,2,*^, Xiaoyu Zhao^1,*^

^1^Shanghai Sci-Tech Inno Center for Infection & Immunity, National Medical Center for Infectious Diseases, Huashan Hospital, Institute of Infection and Health, Shanghai Key Laboratory of Oncology Target Discovery and Antibody Drug Development, Fudan University, Shanghai, China

^2^Shanghai Pudong Hospital, Fudan University Pudong Medical Center, State Key Laboratory of Genetic Engineering, MOE Engineering Research Center of Gene Technology, School of Life Sciences, Shanghai Institute of Infectious Disease and Biosecurity, Fudan University, Shanghai, China

^3^Department of Microbiology, School of Clinical Medicine, Li Ka Shing Faculty of Medicine, The University of Hong Kong, Pokfulam, Hong Kong Special Administrative Region, China

^4^Shanghai Fifth People's Hospital, Shanghai Institute of Infectious Disease and Biosecurity, Institutes of Biomedical Sciences, Fudan University, Shanghai, China.

^5^Department of Infectious Diseases, Shanghai Key Laboratory of Infectious Diseases and Biosafety Emergency Response, National Medical Center for Infectious Diseases, Huashan Hospital, Fudan University, Shanghai, China

^6^School of Public Health, Fudan University, Key Laboratory of Public Health Safety, Ministry of Education, Shanghai, China

^#^These authors contributed equally.

^*^Address correspondence to Xiaoyu Zhao ([xiaoyu_zhao@fudan.edu.cn](mailto:xiaoyu_zhao@fudan.edu.cn)), Pengfei Wang ([pengfei_wang@fudan.edu.cn](mailto:pengfei_wang@fudan.edu.cn)), or Hin Chu ([hinchu@hku.hk](mailto:hinchu@hku.hk)).

**Supplemental Figures**


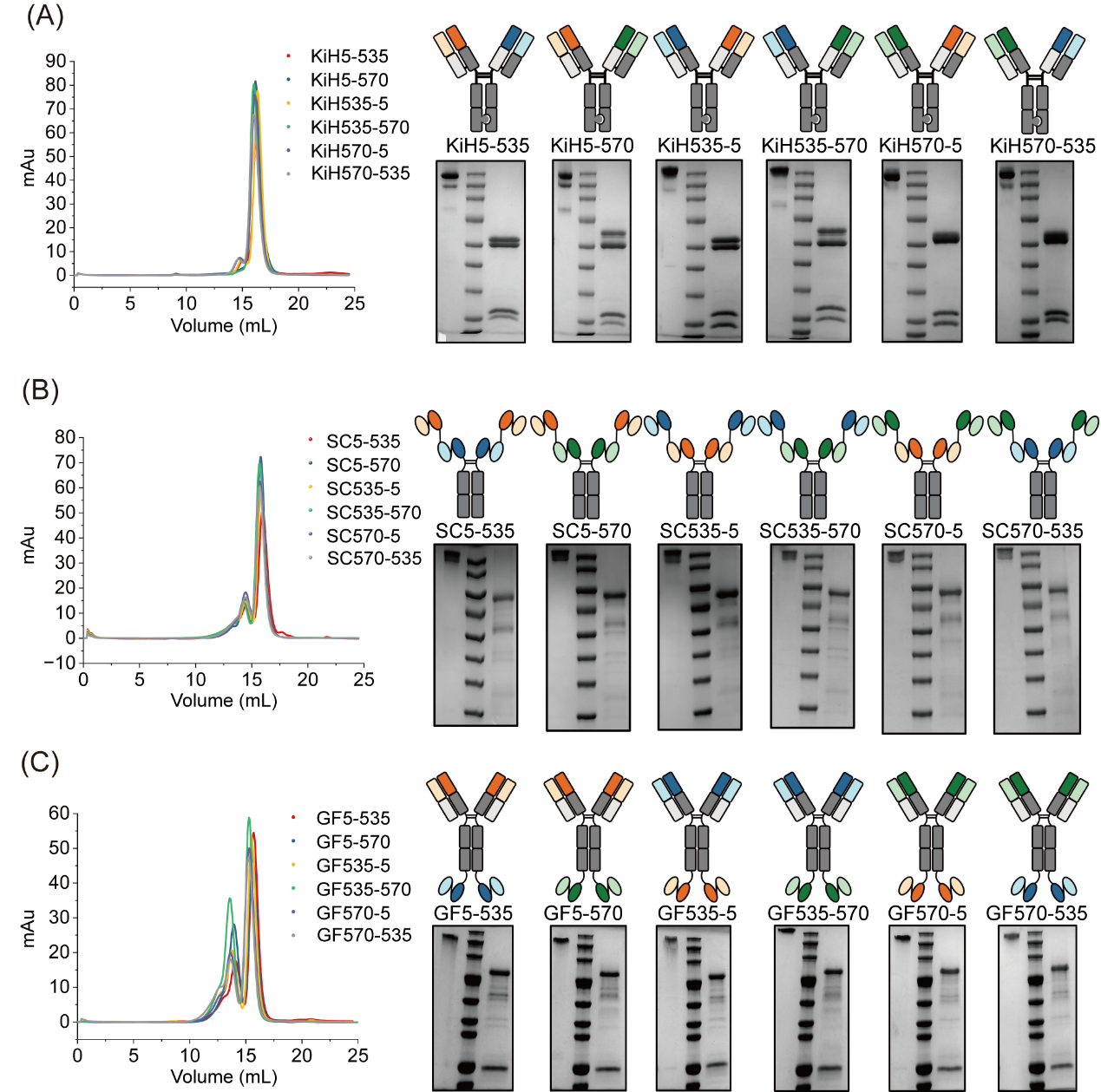


**Figure S1** **Characterization of bispecific antibodies.** Purities of indicated bsAbs with KiH format (**A**), SC format (**B**), or GF format (**C**) were analyzed by SEC, separately, followed by non-reduced (left) and reduced (right) SDS-PAGE analysis to confirm purity. Schematic diagrams illustrating the molecular configurations of bsAbs based on the color design of the prototype antibody, including PW5-5 (orange), PW5-535 (blue), and PW5-570 (green).


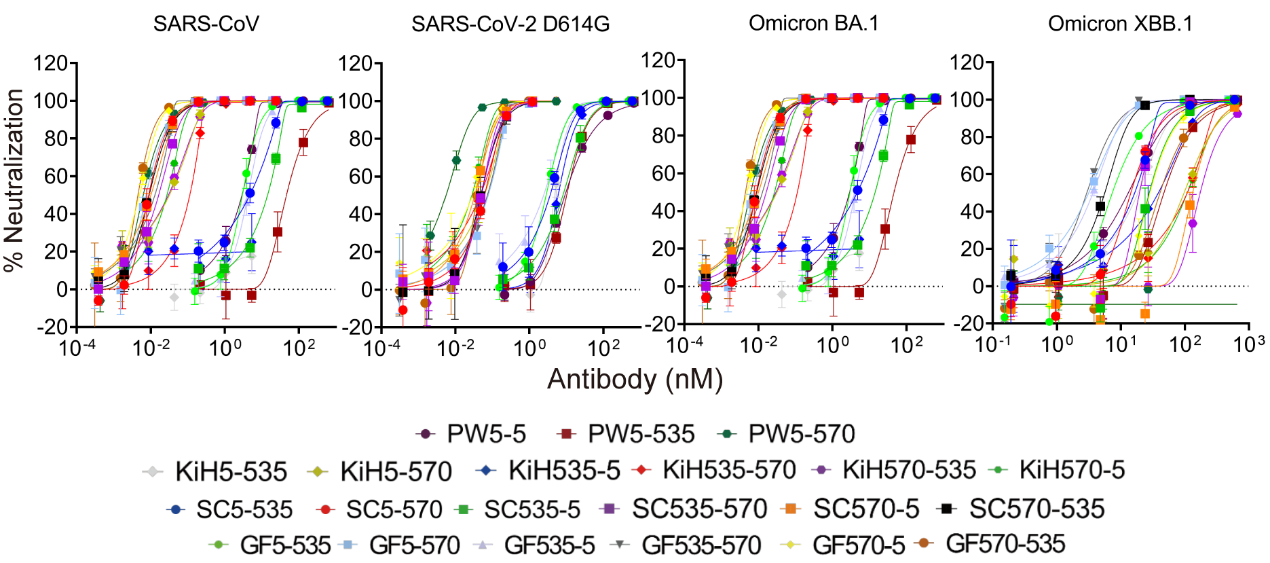


**Figure S2 Neutralization curves using SARS-CoV, SARS-CoV-2 and Omicron variants.** Neutralization curves were conducted in triplicate using the prototype and bsAbs constructs against the SARS-CoV, SARS-CoV-2 WT (D614G), Omicron BA.1 and Omicron XBB.1. The data represent one of at least three independent experiments and are presented as the mean ± SD.


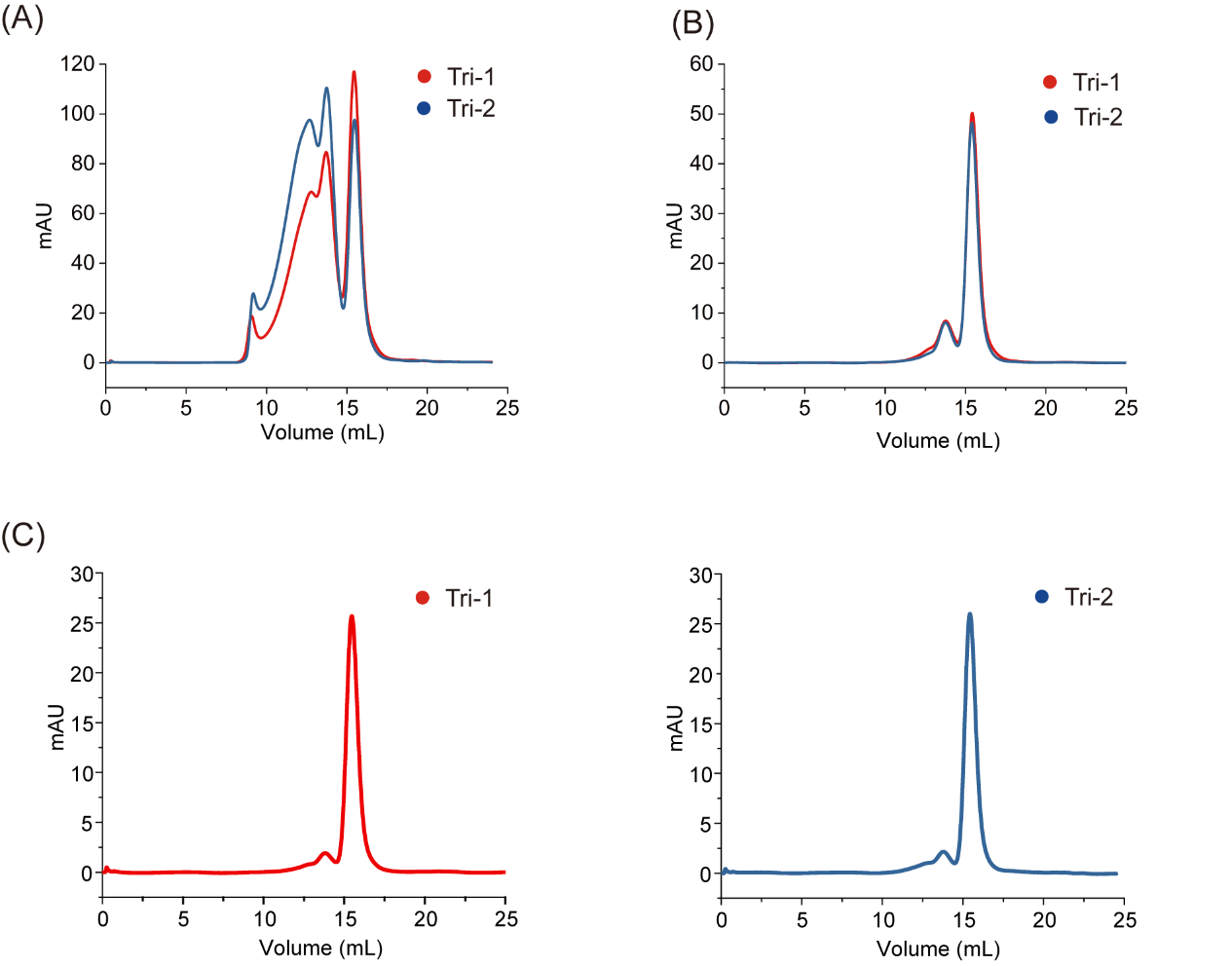


**Figure S3 Purification of trispecific antibodies** **by two-step SEC assay. A-B** The purified trispecific antibodies were analyzed using the ÄKTA pure system with a Superose 6 Increase 10/300 GL column in PBS buffer. **C** The pooled fractions were concentrated, aliquoted, and analyzed by analytical SEC to verify their purity.


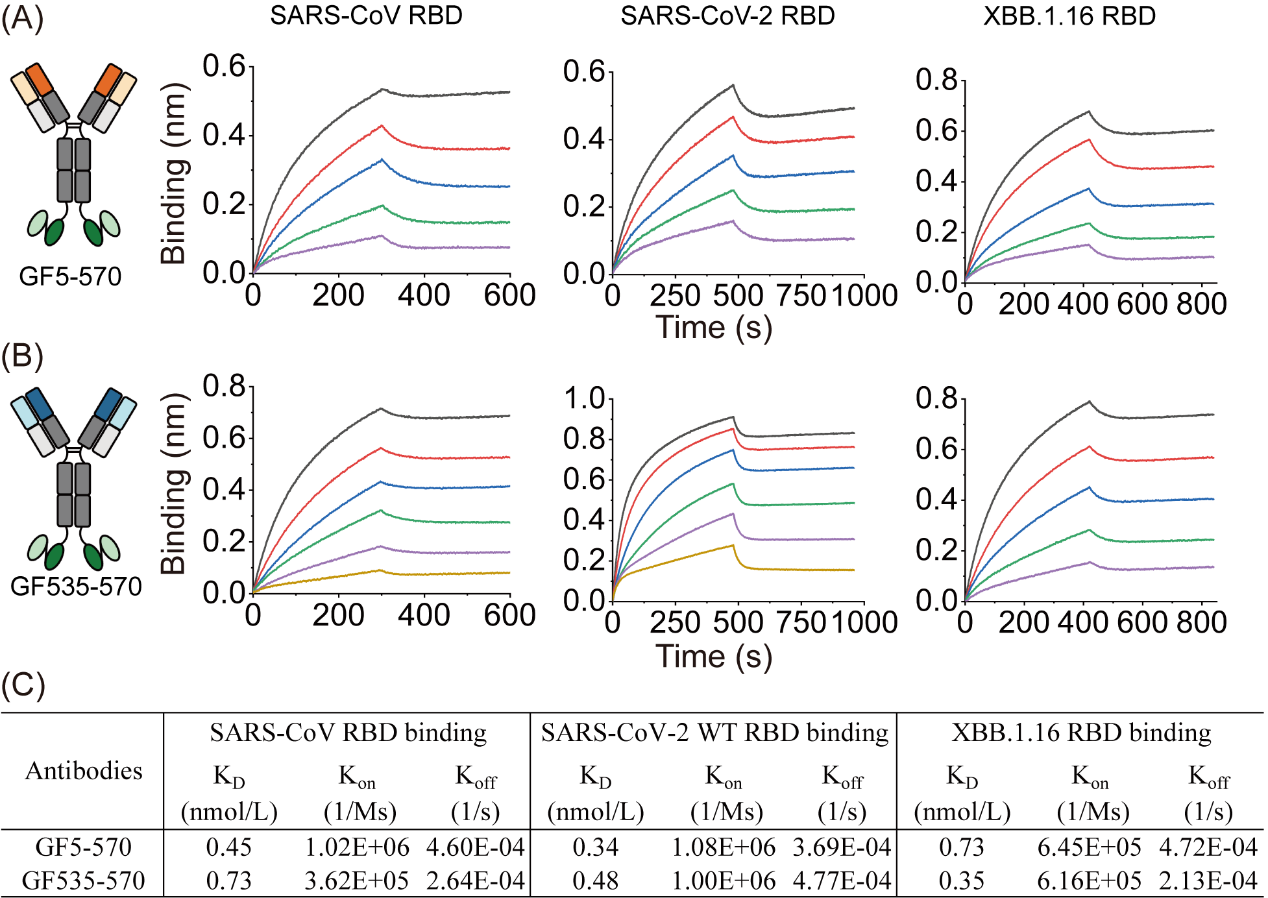


**Figure S4 Binding kinetics of the indicted bsAbs.** Binding kinetics of GF5-570 (**A**) and GF535-570 (**B**) to the RBDs of SARS-CoV, SARS-CoV-2 WT, and XBB.1.16. **C** Summary of the data of the affinities (K_D_), association (K_on_) and dissociation (K_off_) of indicated antibodies as measured by BLI.


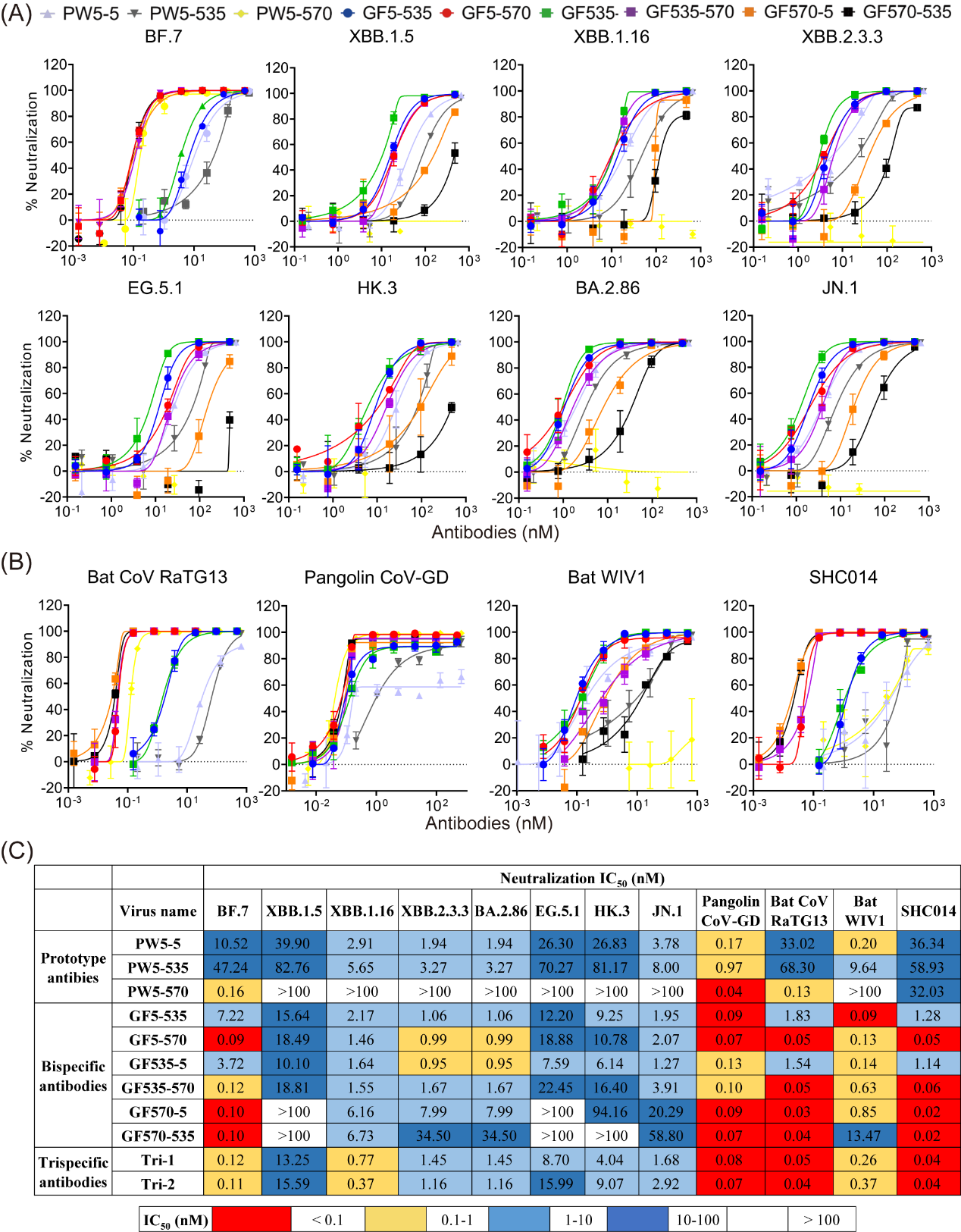


**Figure S5 Neutralization of GF format bsAbs against the current SARS-CoV-2 Omicron variants and other sarbecoviruses. A** Neutralization curves by indicated antibodies against the SARS-CoV-2 Omicron variants, including BF.7, XBB.1.5, XBB.1.16, XBB.2.3.3, BA.2.86, EG.5.1, HK.3 and JN.1. **B** Neutralization curves of SARS-CoV-2 related sarbecoviruses (Pangolin CoV-GD and Bat CoV RaTG13) and SARS-CoV related sarbecoviruses (Bat WIV1 and SHC014) by indicated antibodies. **C** Heatmap with IC_50_ neutralization values of indicated antibodies against SARS-CoV-2 Omicron variants, and other related sarbecovirus pseudoviruses. The data represent one of at least three independent experiments and are presented as the mean ± SD.
